# Anticipating postoperative complications in hepatobiliary surgery: procalcitonine as predictive factor

**DOI:** 10.3389/fsurg.2025.1564843

**Published:** 2025-06-04

**Authors:** Shouwen Zhao, Yuanyuan Song, Le Zhang, Sunan Shi, Jie Li, Yuping Ma, Yong Liao, Zhongguang Zhen

**Affiliations:** ^1^ Department of Hepatobiliary and Pancreatic Surgery, Xingtai People Hospital, Xingtai, Hebei, China; ^2^ Department of Oral and Maxillofacial Surgery, Hebei Provincial Eye Hospital, Xingtai, Hebei, China

**Keywords:** procalcitonin, hepatectomy, postoperative complications, liver failure prognostic factor, liver failure

## Abstract

**Background and aims:**

Few studies have indicated that procalcitonin has a potential role in anticipating postoperative complications of hepatic surgery. Here, we focused on validating the relationship between posthepatectomy and serum procalcitonin as short-term prognostic factors.

**Methods:**

Data from 52 patients who underwent hepatectomy (partial) due to hepatocellular carcinoma from June 2018 to July 2023 were enrolled to evaluate the risk factors related to post-hepatectomy complications, especially post-hepatectomy liver failure and 30-day survival.

**Results:**

52 patients were included in the study, and their data was analyzed for PCT. 21 showed raised PCT (>1 ng/ml). Results showed a significant association of PCT with post-hepatectomy liver failure, and the same is associated with 30-day mortality in ICU-admitted patients.

**Conclusion:**

Elevated PCT levels in patients after hepatic surgery are associated with poor prognosis and could be used as a potential predictive factor.

## 1 Introduction

A 116-amino acid peptide known as procalcitonin (PCT) is produced by the thyroid, liver, lung, kidney, adrenal glands, prostate gland, small intestine, testosterone and adipose tissue in healthy individuals. The average serum level is <0.05 ng ml, and higher levels are a marker for severe bacterial infection (1, 2). Besides this, it has been reported that PCT elevation is also indicative of hepato-biliary issues, mainly hepatitis, liver failure, and cirrhosis. PCT consists of 3 subsections: an amino terminus, which has 57 amino acids; immature calcitonin, having 33 amino acids; and last, calcitonin carboxyl-terminus peptide 1 (CCP-1), which is also known as katacalcin comprises 21 amino acids. Genetically, it is controlled by the calcitonin one gene (CALC-1) located on chromosome 11. The initial product of the CALC-1 gene, i.e., prePCT, undergoes proteolytic cleavage to produce PCT. The PCT is further processed to the mature calcitonin form. Mainly, transcription and translation of the CALC-1 gene are limited to the C-cells of the thyroid gland, but up to a limited extent, expression of CALC-1 is present in other neuroendocrine cells. However, the production of PCT is activated in all parenchymal tissues, such as the liver, kidneys, and lungs, in response to bacterial infection. This production of PCT is signaled by cytokines interleukin-6 (IL-6), tumor necrosis factor-α (TNF-α), and interleukin-1β (IL-β). PCT production is inhibited and negatively regulated by interferon-γ, secreted in response to viral infections. This phenomenon makes PCT a more specific marker for bacterial infection (3–7).

In addition to this, it is also considered a predictor in advanced liver disease, including post-surgical prognostic markers (8–20). For many liver diseases, including liver cancer, resection of the affected part/lobe is considered the treatment of choice (21). In this case, the post-operative level of PCT is helpful in monitoring and management of diseases related to infections, especially bacterial infections. It rises as early as 4 h after surgery and drops after 48–72 h. Previously, it has been reported in several studies that liver injury and surgery are a disease prognosis and infection control marker (22).

**Figure 1:**
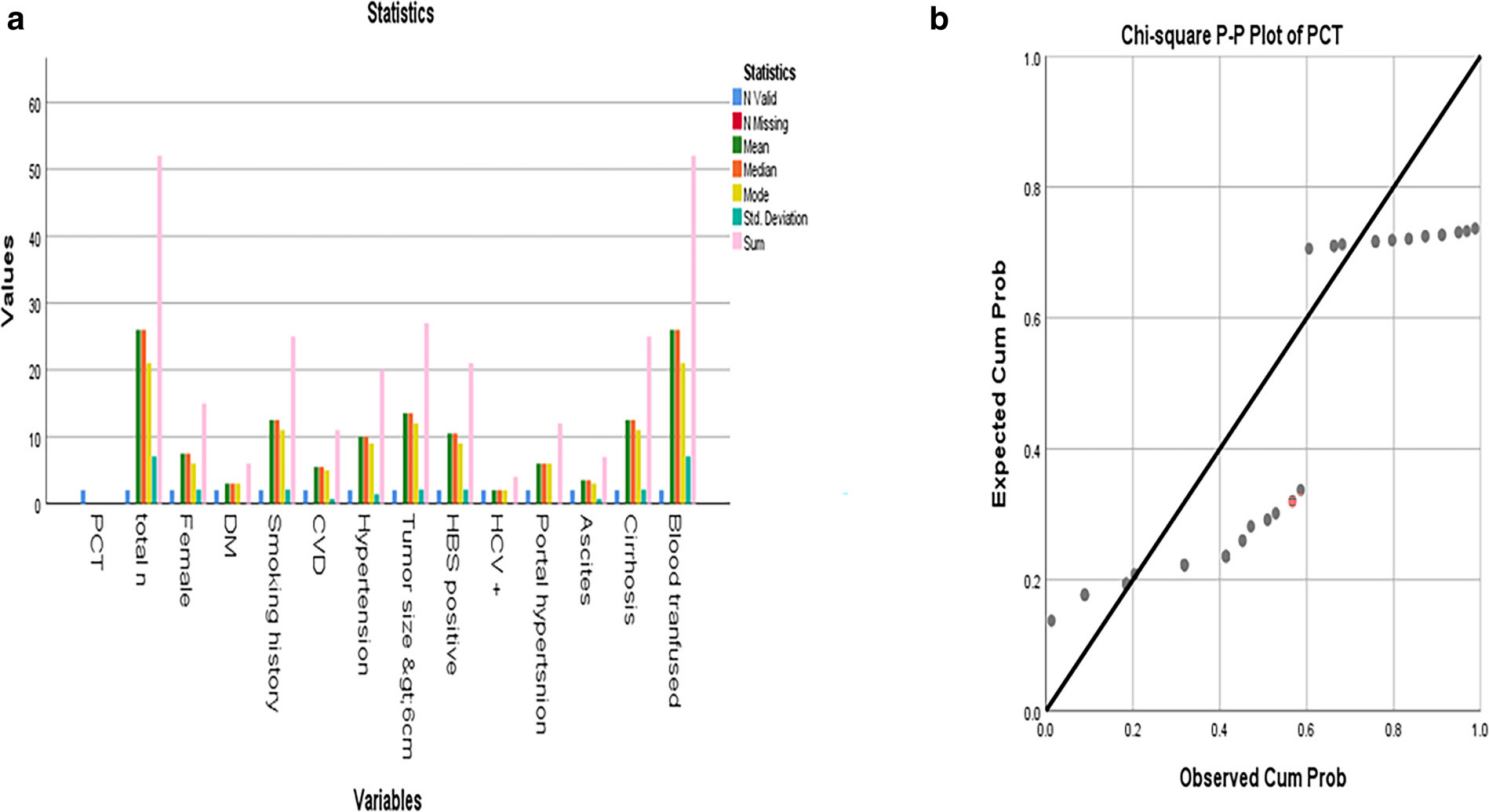
**(a)** Mean, median, and mode of different PCT values among the patients; **(b)** Distribution of PCT levels >1 ng/ml groups. Statistical comparison was performed using the chi-square test.

In the present study, we conducted prognostic value of PCT in patients who underwent liver resection due to liver cancer and its relationship for short-term mortality (30 days) postoperative (Figure 1).

## 2 Materials and methods

We retrospectively identified those eligible patients who underwent hepatectomy from June 2018 to July 2023 at our hospital. The following inclusion criteria were used: (a) patients with HCC who are eligible for hepatectomy/resection; (b) diagnosis was confirmed by histopathology (b) age of 18–80 years; and (c) Child-Pugh A or B. Exclusion criteria were used: (1) patients with a history of multiple metastases (2) patients who have a history of recurrence of disease and previously underwent surgery (3) patients who have a history of any other malignant tumour before hepatectomy; (4) not willing to provide the follow-up information; (5) not willing to provide informed consent. The local ethical committee approved this study, and written informed consent was obtained from all patients.

### 2.1 Data collection

All data reporting is as per currently practicing ethical values and the identity of patients was ensured to remain confidential. The primary outcome of this study was the value of PCT. Other complications, if they happened, are reported in Table. The secondary outcome was taken as mortality within 30 days of the initial operation. The serum PCT levels were measured in serum samples. PCT samples were taken from patients by venous blood drawn after 12 h of surgery and then stored as per standard protocol for immediate reporting at the chemical pathology laboratory of the hospital. A PCT immunoassay was taken (Roche Diagnostics). The minimal detection limit of the assay was 0.05 ng/ml.

All the experiments involved were conducted following ethical standards as approved by Xingtai People's Hospital, 188 Xiangdu Road, Xingtai, China.

### 2.2 Statistical analysis

Continuous variables were presented as mean ± SD or median with IQR (interquartile range). IBM SPSSA 25 is used for this purpose. Measured serum PCT value >1 ng/ml was defined as elevated PCT according to previous research, while each measured PCT value was also applied in the analysis as additional evidence. Mean, median, mode, and other parameters are mentioned in the data—Figures 1a,b.

**Figure 2:**
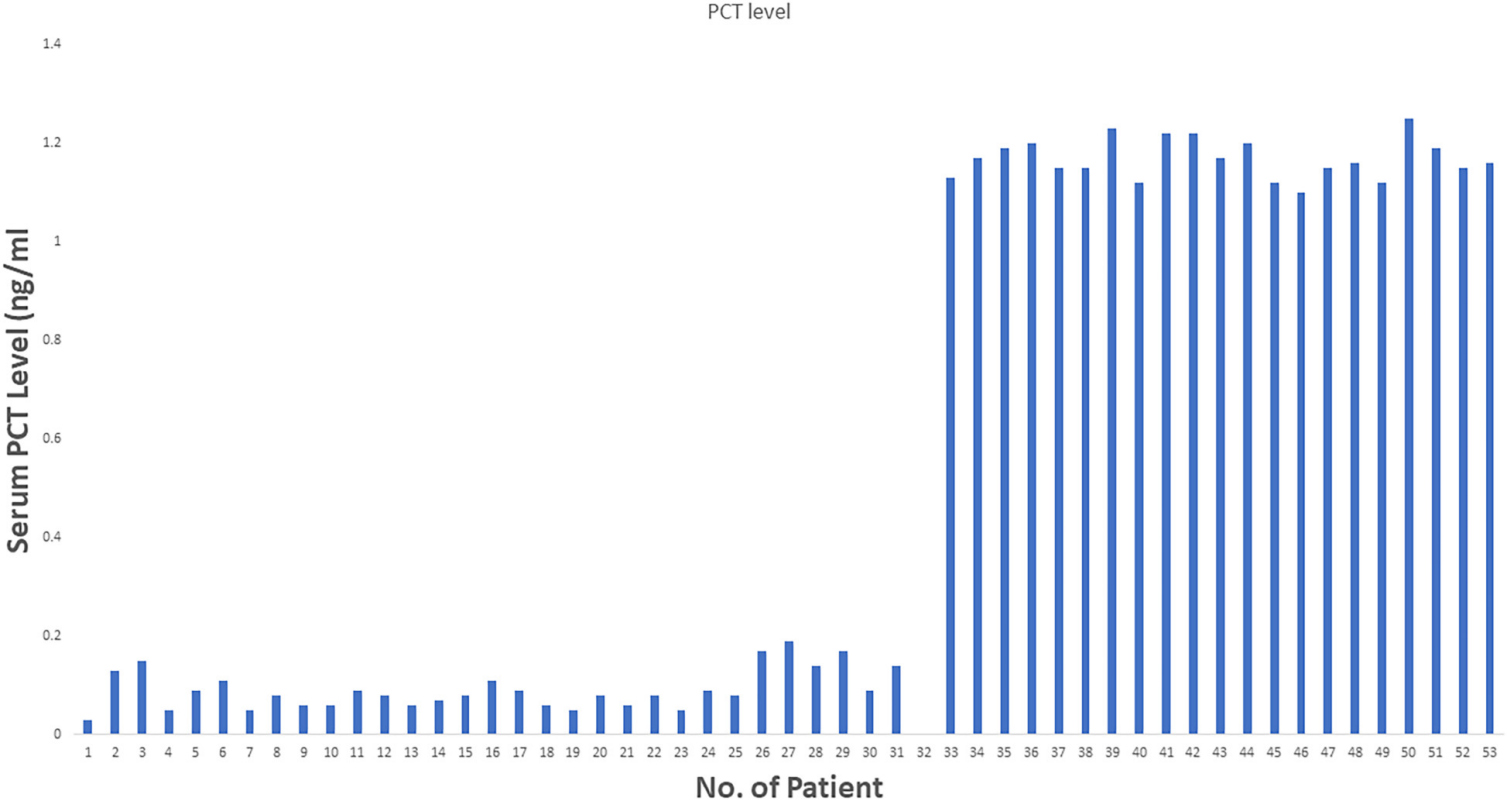
PCT level in all 52 patients.

## 3 Results

**Figure 3:**
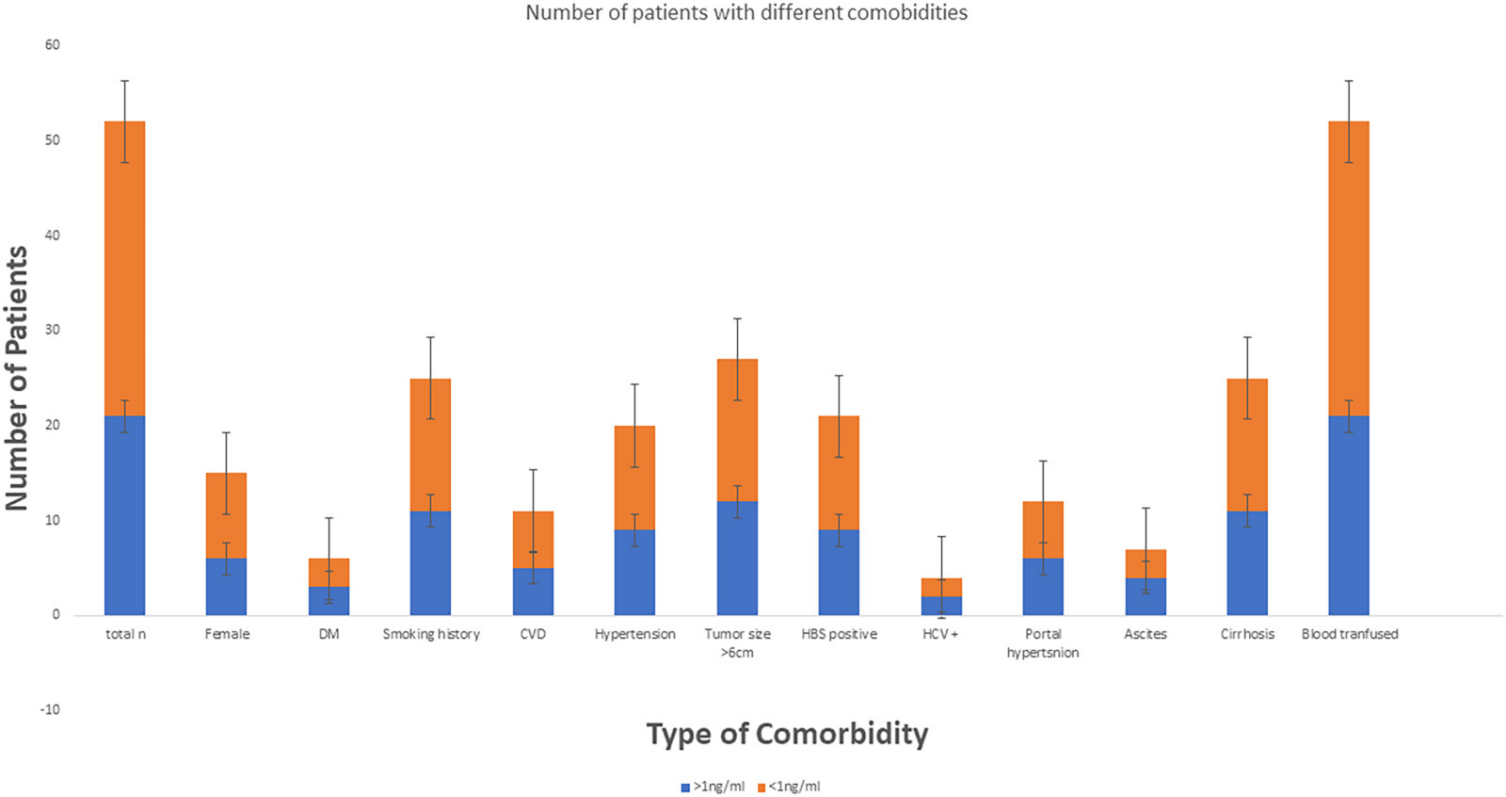
Number of patients with different comorbidities.

**Figure 4:**
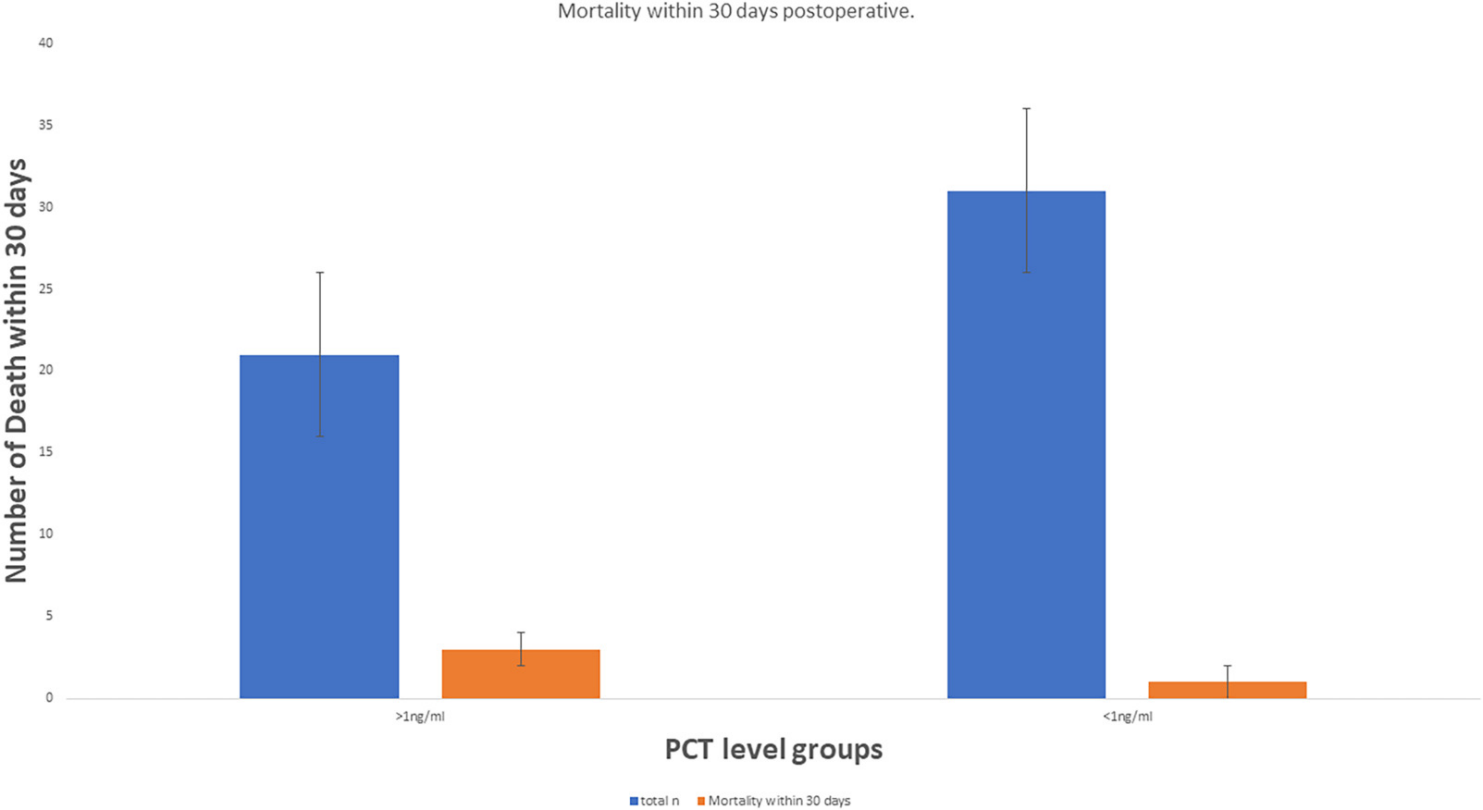
Short-term mortality in both groups.

From June 2018 to July 2023, 52 patients who underwent liver resection for HCC were enrolled in our hospital. The resections included right hepatectomy (*n* = 12), left hepatectomy (*n* = 8), segmentectomy (*n* = 10), bisegmentectomy (*n* = 7), and non-anatomical wedge resection (*n* = 15). After PCT measurement, 31 out of 52 patients had a measured serum PCT level of less than 1 ng/ml, while 21 had reportedly higher serum PCT (≥1 ng/ml). The mean age was 56.51 years. Almost 40.38% of patients showed higher values >1 ng/ml for serum PCT when blood samples were taken after 12 h of surgery (the mean value was 1.169 ng/ml). In addition to this PCT, sex, age, status of diabetes mellitus type 2, history of smoking, history of cardiovascular diseases, hypertension, status of hepatitis B and C positivity, portal hypertension, ascites, and cirrhosis were reported too. The tumor size cut-out value was set as patients having tumor sizes less than 6 cm or more than 6 cm. Blood transfusion was required for all 52 patients, and a maximum of one pint of whole blood was needed. The study parameters are mentioned in Table 1, and in Figure 2, the level of all patients’ PCT is mentioned (Supplementary Table). Different patients having PCT levels <1 ng/ml and >1 ng/ml and their history/relationship with status of diabetes mellitus type 2, history of smoking, history of cardiovascular diseases, hypertension, the status of hepatitis B and C positivity, portal hypertension, ascites, and cirrhosis is mentioned in Table 2 and Figure 3, respectively. In Table 3, patients with serum PCT levels <1 ng/ml and >1 ng/ml and their secondary outcome, i.e., mortality within 30 days, are also shown. The group of patients who had serum PCT levels less than 1 ng/ml had one reported death (3.22) within 30 days after surgery. At the same time, the group of patients who had serum PCT levels of more than 1 ng/ml had 3 (14.28) deaths within 30 days of surgery. It showed a significant difference between the secondary outcome, i.e., mortality within 30 days of surgery (Figure 4).

**Table 1:** Clinical and demographic profile of patients with PCT <1 ng/ml.

Group <1 ng/ml	PCT	Sex	Age	DM	Smoking history	CVD	Hypertension	Tumor size >6 cm	HBS positive	HCV +	Portal hypertsnion	Ascites	Cirrhosis
	0.03	F	57	No	Yes	Yes	Yes	Yes	Yes	No	Yes	No	Yes
	0.13	M	48	Yes	Yes	No	No	No	No	Yes	Yes	Yes	Yes
	0.15	F	76	No	No	No	No	No	No	No	No	No	No
	0.05	M	44	No	Yes	Yes	Yes	Yes	Yes	No	Yes	No	Yes
	0.09	M	53	Yes	Yes	Yes	Yes	Yes	Yes	No	No	No	Yes
	0.11	F	60	No	No	No	No	No	No	No	No	No	No
	0.05	M	54	No	No	No	No	No	No	No	No	No	No
	0.08	M	64	No	No	No	No	No	Yes	No	No	No	Yes
	0.06	M	49	No	No	No	No	No	Yes	No	Yes	No	Yes
	0.06	M	55	Yes	Yes	Yes	Yes	Yes	Yes	No	No	No	No
	0.09	M	67	No	No	No	No	No	No	Yes	Yes	Yes	No
	0.08	M	55	No	No	No	No	No	No	No	No	No	Yes
	0.06	M	51	No	No	No	No	No	No	No	No	No	Yes
	0.07	F	76	No	No	No	No	No	No	No	No	No	No
	0.08	M	54	No	Yes	Yes	Yes	Yes	Yes	No	Yes	Yes	Yes
	0.11	M	53	No	No	No	No	No	No	No	No	No	No
	0.09	F	71	No	No	No	No	No	No	No	No	No	No
	0.06	F	55	No	Yes	No	Yes	Yes	No	No	No	No	No
	0.05	M	54	No	No	No	No	No	No	No	No	No	Yes
	0.08	M	55	No	No	No	No	No	Yes	No	No	No	Yes
	0.06	M	43	No	Yes	No	Yes	Yes	No	No	No	No	No
	0.08	F	57	No	Yes	Yes	Yes	Yes	No	No	No	No	No
	0.05	M	65	No	No	No	No	No	Yes	No	No	No	Yes
	0.09	M	45	No	Yes	No	Yes	Yes	No	No	No	No	No
	0.08	F	43	No	No	No	No	No	Yes	No	No	No	Yes
	0.17	M	45	No	No	No	No	Yes	No	No	No	No	Yes
	0.19	M	43	No	No	No	No	No	Yes	No	No	No	No
	0.14	M	45	No	No	No	Yes	Yes	No	No	No	No	No
	0.17	M	56	No	No	No	Yes	Yes	No	No	No	No	No
	0.09	M	76	No	No	No	No	No	Yes	No	No	No	No
	0.14	F	54	No	No	No	No	No	No	No	No	No	No
Group >1 ng/ml													
	1.13	F	55	No	No	No	No	No	No	No	No	No	No
	1.17	M	61	No	Yes	Yes	Yes	Yes	Yes	No	No	No	Yes
	1.19	F	55	No	No	No	No	No	No	Yes	Yes	Yes	No
	1.2	M	56	No	Yes	No	Yes	Yes	Yes	No	No	No	No
	1.15	M	52	No	No	No	No	No	Yes	No	Yes	No	No
	1.15	F	66	Yes	Yes	Yes	Yes	Yes	Yes	No	Yes	No	No
	1.23	F	67	No	Yes	Yes	Yes	Yes	Yes	No	No	No	No
	1.12	M	45	No	No	No	No	No	No	No	No	No	Yes
	1.22	M	65	Yes	No	No	No	No	Yes	No	Yes	Yes	No
	1.22	M	45	No	No	No	No	Yes	Yes	No	No	No	Yes
	1.17	F	54	No	No	No	No	No	No	No	No	Yes	Yes
	1.2	M	54	No	No	No	No	No	No	Yes	Yes	Yes	Yes
	1.12	F	65	No	Yes	Yes	Yes	Yes	No	No	No	No	Yes
	1.1	M	56	Yes	No	No	No	No	Yes	No	Yes	No	No
	1.15	M	72	No	Yes	Yes	Yes	Yes	No	No	No	No	No
	1.16	M	71	No	Yes	No	Yes	Yes	No	No	No	No	No
	1.12	M	54	No	No	No	No	No	No	No	No	No	Yes
	1.25	M	64	No	Yes	No	Yes	Yes	Yes	No	No	No	Yes
	1.19	M	56	No	No	No	No	Yes	No	No	No	No	Yes
	1.15	M	60	No	Yes	No	Yes	Yes	No	No	No	No	Yes
	1.16	M	43	No	No	No	No	Yes	No	No	No	No	Yes

**Table 2:** Clinical profile and comorbidities of patients by PCT levels.

PCT	Total n	Female	DM	Smoking history	CVD	Hypertension	Tumor size >6 cm	HBS positive	HCV+	Portal hypertsnion	Ascites	Cirrhosis
>1 ng/ml	21	6	3	11	5	9	12	9	2	6	4	11
<1 ng/ml	31	9	3	14	6	11	15	12	2	6	3	14
*p*-value		1.000	0.946	0.819	0.968	0.806	0.736	0.991	1.000	0.661	0.577	0.819

**Table 3:** 30-Day mortality rates and percentages based on procalcitonin (PCT) levels.

PCT	total *n*	Mortality within 30 days	Percentage	*p*-value
>1 ng/ml	21	3	14.28%	0.348
<1 ng/ml	31	1	3.22%	

## 4 Discussion

PCT is a marker of various bacterial infections and a proven prognostic marker. In addition to this, few other authors have already reported the role of PCT in liver disease. Based on the findings that PCT is a prognostic marker (3–7) for various diseases, we chose it for our study. Here we showed that along with other factors, PCT level >1 ng/ml has a significant role (mean 1.196 ng/ml), especially in short-term mortality. In the patient group with PCT level >1 ng/ml, 14.28% mortality within 30 postoperative days is reported. While in the patient group, which reported an initial level of PCT <1 ng/ml, only 3.22% mortality in 30 days was reported (mean PCT level 0.091 ng/ml). It shows that PCT is valuable for anticipating prognostic factors for surgical outcomes for liver surgery patients. In addition, patients with cirrhosis have a greater chance of bacterial infection (23). Jekerl et al. (24) also reported that the value of PCT increased two days after abdominal surgery, which correlated to systemic infection risk. Moreover, persistently raised levels of PCT for two consecutive days post-surgically are related to unfavorable outcomes (25).

The shorter half-life of PCT, which is maximally up to 24 h, makes PCT an essential marker in this context. If, for two days, values of PCT are raised or maintained, then it shows infection involvement is at a systemic level. Previously few studies have demonstrated the relationship of PCT with liver surgery and outcomes (26, 27). Several complications, like portal hypertension, ascites, and previous hepatitis status, were linked to higher levels of PCT. Previously, it has been shown that alcoholic liver cirrhosis and portal hypertension induced increased levels of PCT. Although PCT's origin in the human body was initially traced to thyroid tissue, it was later proved to be produced by the liver. So, in this case, an injury or surgery of the liver plays a vital role in the rise of PCT, especially within the first 48 h. These liver-associated complications, like portal hypertension, have an impact on bacterial translocation by increasing intestinal permeability, and studies have shown that patients with cirrhosis and ascites have higher rates of bacterial infections as compared to controls. Moreover, these bacterial strains are more resistant to conventional antibiotics. This evidence and potential mechanism show that PCT indicates the initial stage of bacteremia, which subsequently leads to sepsis. The same is the case in our study. Two patients died of sepsis within 30 days post-surgery, and both of these patients had initial PCT levels > 1 ng/ml. In addition to sepsis, previous studies show that elevated levels of PCT are indicative of severe pancreatitis, renal dysfunction including renal failure, cardiogenic hypovolemic shock, and in all these complications, it is associated with poor prognosis.

Endotoxin, a lipopolysaccharide present in gram-negative bacteria, is considered to play its role in the increase of PCT by Toll-like receptor four, which is a transmembrane receptor expressed on different immune cells like neutrophils, macrophages, monocytes, and dendritic cells. On activation of these immune cells, mediators like tumor necrosis factor (TNF) and interleukin one beta, 6, 8, and 10 are produced, which leads to PCT secretion by cytokine-activated macrophages from different organs mentioned earlier (2). So, like other inflammatory markers, PCT is an emerging marker for different severe medical and surgical conditions. In our study, PCT value has a link with other complications, including sepsis and cirrhosis etches, which was the cause of death in 2 patients 30 days postoperative who had an initial value of PCT >1 ng/ml. It proves that involvement of the immune system and clinical management of the patient has the potential role of elevated levels of PCT.

PCT level has also been linked to other clinical fields, such as a few cancers. Solid tumors (like thyroid carcinoma) and a few hematological malignancies are linked positively with raised levels of PCT (28, 29). C-cells of the thyroid are the primary site of PCT production, and in other neuroendocrine cells, a spontaneous PCT increase in neuroendocrine cells could be elevated. Some authors reported that PCT could better discriminate infections and para-neoplastic fever (28, 30). Liver cancer, especially hepatocellular carcinoma, also affects various hormone/peptide levels *in vivo* because the liver is also an endocrine organ. Therefore, it is more apparent in the case of hepatic surgery how PCT plays a role in prognosis and mortality (31). In addition, the PCT level is proportionately linked to the mortality rate in the case of liver disorders. Lu et al. reported that a high level of PCT was related to poor prognosis in the case of cirrhosis who was suffering from hepatic carcinoma (32). It showed that PCT has a growing exploratory role in the case of liver disorders, especially hepatic cancer patients. This opens the path for future exploration to understand better and link PCT with liver diseases beyond the role as a marker for bacterial infection (33–37).

## 5 Conclusion

This study shows that PCT is a potential anticipating marker in liver surgery, and its monitoring could guide clinical management and, hence, postoperative outcomes. In addition, PCT's role for patients who may develop subsequent sepsis if initial serum levels are raised could also be a valuable guide for selecting appropriate antibacterial medication based on blood culture and sensitivity.

## Data Availability

The original contributions presented in the study are included in the article/Supplementary Material, further inquiries can be directed to the corresponding author.
