# Supplementary material for: Anticipating postoperative complications in hepatobiliary surgery: procalcitonine as predictive factor
Source: Front Surg. 2025 Jun 4;12:1564843. doi: 10.3389/fsurg.2025.1564843 (PMC12173904; doi:10.3389/fsurg.2025.1564843)
Supplement: Supplementary file 1 [file Table1.docx]

**Supplementary Table**

| Group <1ng/ml | | PCT | sex | age | DM | Smoking history | CVD | Hypertension | Tumor size >6cm | HBS positive | HCV + | Portal hypertsnion | Ascites | Cirrhosis |
| --- | --- | --- | --- | --- | --- | --- | --- | --- | --- | --- | --- | --- | --- | --- |
|  | 0.03 | | F | 57 | No | Yes | Yes | Yes | Yes | Yes | No | Yes | No | Yes |
|  | 0.13 | | M | 48 | Yes | Yes | No | No | No | No | Yes | Yes | Yes | Yes |
|  | 0.15 | | F | 76 | No | No | No | No | No | No | No | No | No | No |
|  | 0.05 | | M | 44 | No | Yes | Yes | Yes | Yes | Yes | No | Yes | No | Yes |
|  | 0.09 | | M | 53 | Yes | Yes | Yes | Yes | Yes | Yes | No | No | No | Yes |
|  | 0.11 | | F | 60 | No | No | No | No | No | No | No | No | No | No |
|  | 0.05 | | M | 54 | No | No | No | No | No | No | No | No | No | No |
|  | 0.08 | | M | 64 | No | No | No | No | No | Yes | No | No | No | Yes |
|  | 0.06 | | M | 49 | No | No | No | No | No | Yes | No | Yes | No | Yes |
|  | 0.06 | | M | 55 | Yes | Yes | Yes | Yes | Yes | Yes | No | No | No | No |
|  | 0.09 | | M | 67 | No | No | No | No | No | No | Yes | Yes | Yes | No |
|  | 0.08 | | M | 55 | No | No | No | No | No | No | No | No | No | Yes |
|  | 0.06 | | M | 51 | No | No | No | No | No | No | No | No | No | Yes |
|  | 0.07 | | F | 76 | No | No | No | No | No | No | No | No | No | No |
|  | 0.08 | | M | 54 | No | Yes | Yes | Yes | Yes | Yes | No | Yes | Yes | Yes |
|  | 0.11 | | M | 53 | No | No | No | No | No | No | No | No | No | No |
|  | 0.09 | | F | 71 | No | No | No | No | No | No | No | No | No | No |
|  | 0.06 | | F | 55 | No | Yes | No | Yes | Yes | No | No | No | No | No |
|  | 0.05 | | M | 54 | No | No | No | No | No | No | No | No | No | Yes |
|  | 0.08 | | M | 55 | No | No | No | No | No | Yes | No | No | No | Yes |
|  | 0.06 | | M | 43 | No | Yes | No | Yes | Yes | No | No | No | No | No |
|  | 0.08 | | F | 57 | No | Yes | Yes | Yes | Yes | No | No | No | No | No |
|  | 0.05 | | M | 65 | No | No | No | No | No | Yes | No | No | No | Yes |
|  | 0.09 | | M | 45 | No | Yes | No | Yes | Yes | No | No | No | No | No |
|  | 0.08 | | F | 43 | No | No | No | No | No | Yes | No | No | No | Yes |
|  | 0.17 | | M | 45 | No | No | No | No | Yes | No | No | No | No | Yes |
|  | 0.19 | | M | 43 | No | No | No | No | No | Yes | No | No | No | No |
|  | 0.14 | | M | 45 | No | No | No | Yes | Yes | No | No | No | No | No |
|  | 0.17 | | M | 56 | No | No | No | Yes | Yes | No | No | No | No | No |
|  | 0.09 | | M | 76 | No | No | No | No | No | Yes | No | No | No | No |
|  | 0.14 | | F | 54 | No | No | No | No | No | No | No | No | No | No |
| Group >1ng/ml |  | |  |  |  |  |  |  |  |  |  |  |  |  |
|  | 1.13 | | F | 55 | No | No | No | No | No | No | No | No | No | No |
|  | 1.17 | | M | 61 | No | Yes | Yes | Yes | Yes | Yes | No | No | No | Yes |
|  | 1.19 | | F | 55 | No | No | No | No | No | No | Yes | Yes | Yes | No |
|  | 1.2 | | M | 56 | No | Yes | No | Yes | Yes | Yes | No | No | No | No |
|  | 1.15 | | M | 52 | No | No | No | No | No | Yes | No | Yes | No | No |
|  | 1.15 | | F | 66 | Yes | Yes | Yes | Yes | Yes | Yes | No | Yes | No | No |
|  | 1.23 | | F | 67 | No | Yes | Yes | Yes | Yes | Yes | No | No | No | No |
|  | 1.12 | | M | 45 | No | No | No | No | No | No | No | No | No | Yes |
|  | 1.22 | | M | 65 | Yes | No | No | No | No | Yes | No | Yes | Yes | No |
|  | 1.22 | | M | 45 | No | No | No | No | Yes | Yes | No | No | No | Yes |
|  | 1.17 | | F | 54 | No | No | No | No | No | No | No | No | Yes | Yes |
|  | 1.2 | | M | 54 | No | No | No | No | No | No | Yes | Yes | Yes | Yes |
|  | 1.12 | | F | 65 | No | Yes | Yes | Yes | Yes | No | No | No | No | Yes |
|  | 1.1 | | M | 56 | Yes | No | No | No | No | Yes | No | Yes | No | No |
|  | 1.15 | | M | 72 | No | Yes | Yes | Yes | Yes | No | No | No | No | No |
|  | 1.16 | | M | 71 | No | Yes | No | Yes | Yes | No | No | No | No | No |
|  | 1.12 | | M | 54 | No | No | No | No | No | No | No | No | No | Yes |
|  | 1.25 | | M | 64 | No | Yes | No | Yes | Yes | Yes | No | No | No | Yes |
|  | 1.19 | | M | 56 | No | No | No | No | Yes | No | No | No | No | Yes |
|  | 1.15 | | M | 60 | No | Yes | No | Yes | Yes | No | No | No | No | Yes |
|  | 1.16 | | M | 43 | No | No | No | No | Yes | No | No | No | No | Yes |
